# Results of ARI-0001 CART19 Cells in Patients With Chronic Lymphocytic Leukemia and Richter’s Transformation

**DOI:** 10.3389/fonc.2022.828471

**Published:** 2022-01-31

**Authors:** Valentín Ortiz-Maldonado, Gerard Frigola, Marta Español-Rego, Olga Balagué, Nuria Martínez-Cibrián, Laura Magnano, Eva Giné, Mariona Pascal, Juan G. Correa, Alexandra Martínez-Roca, Joan Cid, Miquel Lozano, Neus Villamor, Daniel Benítez-Ribas, Jordi Esteve, Armando López-Guillermo, Elías Campo, Álvaro Urbano-Ispizua, Manel Juan, Julio Delgado

**Affiliations:** ^1^ Department of Hematology, Hospital Clínic de Barcelona, Barcelona, Spain; ^2^ Oncology and Hematology, Institut d’Investigacions Biomèdiques August Pi i Sunyer (IDIBAPS), Barcelona, Spain; ^3^ Department of Pathology, Hospital Clínic de Barcelona, Barcelona, Spain; ^4^ Department of Immunology, Hospital Clínic de Barcelona, Barcelona, Spain; ^5^ Centro de Investigación Biomédica en Red de Cáncer (CIBERONC), Barcelona, Spain; ^6^ Apheresis & Cell Therapy Unit, Department of Hemotherapy and Hemostasis, Hospital Clínic de Barcelona, Barcelona, Spain; ^7^ Department of Medicine, University of Barcelona, Barcelona, Spain; ^8^ Hematopathology Unit, Hospital Clínic de Barcelona, Barcelona, Spain; ^9^ Stem Cell Transplant and Cell Immunotherapy Group, Institute of Research Josep Carreras, Barcelona, Spain

**Keywords:** CLL, CART, CD19, DLBCL, Richter disease

## Abstract

CART19 cells are emerging as an alternative therapy for patients with chronic lymphocytic leukemia (CLL). Here we report the outcome of nine consecutive patients with CLL treated with ARI-0001 CART19 cells, six of them with Richter’s transformation (RT). One patient with RT never received therapy. The cytokine release syndrome rate was 87.5% (12.5% grade ≥3). Neurotoxicity was not observed in any patient. All patients experienced absolute B-cell aplasia, and seven (87.5%) responded to therapy. With a median follow-up of 5.6 months, two patients with RT experienced a CD19-negative relapse. In conclusion, ARI-0001 cell therapy was feasible, safe, and effective in patients with high-risk CLL or RT.

## Introduction

Patients with chronic lymphocytic leukemia (CLL) who do not respond to targeted therapies have an unfavourable prognosis, particularly if tumor cells harbour high-risk genomic aberrations (e.g. *TP53* aberrations or complex karyotype) or the disease has transformed into diffuse large B-cell lymphoma (DLBCL), also known as Richter’s transformation (RT) (1). Current guidelines recommend allogeneic hematopoietic cell transplantation (alloHCT) for patients with high-risk CLL or RT (1–3). Chimeric antigen receptor T-cells targeting CD19 (CART19) are also emerging as alternative options for these patients, even though there are currently no approved products for them.

At Hospital Clinic of Barcelona, we have developed our own CART19 product (A3B1:CD8:41BB : CD3z or ARI-0001), which has been recently approved by the Spanish Medicines Agency (AEMPS) for the treatment of patients with relapsed/refractory acute lymphoblastic leukemia (ALL) older than 25 years of age (4).

The aim of this paper is to report the outcome of all consecutive patients with CLL treated with ARI-0001 cells, either within the CART19-BE-01 trial or a subsequent compassionate use program (CUP).

## Methods

### Patient Eligibility and Clinical Trial Design

ARI-0001 cells are autologous T-cells transduced with a CD137-based second generation CAR construct designed to target CD19 (5). Full details of the ARI-0001 cell development, including its structure and phenotypic characteristics can be found elsewhere (5, 6). Two patients presented here were recruited into the CART19-BE-01 study (registered as NCT03144583) (7), and the remaining patients were included in a CUP with the same inclusion criteria: (i) CD19-positive B-cell malignancy (including DLBCL or CLL); (ii) age from 2 to 80 years; (iii) ECOG performance status 0-2; (iv) estimated life expectancy from 3 months to 2 years; and (v) adequate venous access (7). Patients with CLL were eligible if they had received a minimum of 2 lines of therapy, including rituximab, and experienced disease progression within 2 years of last therapy. Key exclusion criteria included history of other malignancy unless it had been in remission for more than 3 years; severe renal, hepatic, pulmonary or cardiac impairment; active immunosuppressive therapy; HIV infection; active HBV or HCV infection; and active infection requiring systemic therapy. Of note, neither central nervous system involvement nor prior alloHCT were exclusion criteria for this trial.

Whenever feasible, patients with CLL/RT were asked to take ibrutinib, at the approved dose or less in case of intolerance, for a minimum of two weeks until leukocytapheresis. Before ARI-0001 cell infusion, patients received fludarabine at 30 mg/m^2^/day plus cyclophosphamide at 300 mg/m^2^/day on days −6, −5, and −4 followed by ARI-0001 cells. The first patient received a single intravenous infusion of ARI-0001 cells, at a dose of 1 ×10^6^ cells/kg, on day 0. The remaining patients received a fractionated target dose of 1 ×10^6^ cells/kg (patients with CLL only) or 5 ×10^6^ cells/kg (patients with RT). The first fraction (10%) of ARI-0001 cells was administered on day 0, followed by the second (30%) and third (60%) fraction 24-48 hours after the first and second fraction, respectively, if the patient had no signs or symptoms of cytokine release syndrome (CRS). The implementation of the fractionated administration of ARI-0001 cells was motivated by 3 toxic deaths, all in patients with ALL (7). Intravenous immunoglobulin (Ig) replacement was recommended in case of IgG determinations lower than 4 g/L.

All patients provided written, informed consent. The AEMPS and our Institutional Review Board approved the trial, which was conducted in accordance with the principles of the Declaration of Helsinki (last updated version, Fortaleza, Brazil, 2013).

### Measurement of ARI-0001 Cells and CAR19 Transgene

ARI-0001 cells presence was evaluated by flow cytometry with an APC-conjugated AffiniPureF(ab’)_2_-fragment goat-anti-mouse IgG monoclonal antibody (goat-anti-mouse IgG, Jackson ImmunoResearch Laboratories). Moreover, a quantitative PCR assay was optimized and validated for monitoring ARI-0001 cell expansion and persistence. The number of transgene copies/cell was determined by quantitative real-time PCR, using Light Cycler^®^ 480 SYBRGreen^®^ I Master (Roche, Cat. N. 04707516001). Pairs of primers were designed against the GATA2 gene (control) and WPRE sequence (part of the transgene). Primer sequences are as follows: GATA2_F: 5’tggcgcacaactacatggaa 3’; GATA2_R: 5’cgagtcgaggtgattgaagaaga 3’; WPRE_F: 5’gtcctttccatggctgctc 3’; WPRE_R: 5’ccgaagggacgtagcaga 3’. The absolute quantification method was used to determine copy number. Standard curves were prepared using 1:10 serial dilutions of plasmids containing GATA2 or transgene. The final number of molecules in the reaction ranged from 10^2^ to 10^8^ molecules. For GATA2 quantification, GATA2 cDNA was cloned in a pCRII-Topo vector (Invitrogen). pCCL-CAR19 vector was used in the same way to quantify transgene copy number. The following PCR program was used: 1) Initial denaturalization: 95°C, 5’; 2) 40 cycles of: 95°C, 10’’; 58°C, 10’’; 72°C, 5’’; 3) melting curve.

### Endpoints and Statistical Analysis

The primary endpoint of the CART19-BE-01 trial was safety as determined by procedure-related mortality (PRM) and grade 3-4 toxicity at day +100 and one year. Adverse events of special interest were cytokine release syndrome (CRS), neurotoxicity [currently known as immune effector-cell associated neurotoxicity syndrome (ICANS)] and second primary malignancies. Adverse events were graded according to common terminology criteria (CTC), version 4.0. CRS was originally graded as per Lee et al. (8), but was later retrospectively reassessed using ASTCT criteria (9).

Secondary endpoints were objective response rate (ORR) and complete response rate (CRR) at day +28 and +100, duration of response (DOR), duration of B-cell aplasia (DBCA), progression-free survival (PFS) and overall survival (OS). ORR/CRR were assessed as per iwCLL (10) and Lugano (11) criteria as appropriate. Measurable residual disease (MRD) was determined in peripheral blood and bone marrow by flow cytometry, with a sensitivity of 10^-4^.

Adverse events and response rates are presented with 95% exact Clopper-Pearson confidence intervals. OS, PFS, DOR and DBCA, were plotted using the Kaplan-Meier method. Due to the open-label non-randomized nature of the study, the statistical analysis was descriptive and no formal comparisons between cohorts are provided. Statistical analyses were performed using SAS version 9.4 (SAS Institute, Cary, NC) and R (R Foundation for Statistical Computing, Vienna, Austria). The trial (EUDRA n° 2016-2972-29) was registered at clinicaltrials.gov (NCT03144583).

## Results

We report the outcome of nine consecutive patients with CLL treated with ARI-0001 cells, six of them with concomitant RT (**Table 1**). In patients with RT, tumor histology was consistent with DLBCL in patients 2-5 and 8, and plasmablastic lymphoma in patient 6. The median age was 58 (range: 47-74) years, with a male/female ratio of 56/44%. All patients had high-risk progressive disease (2, 3), with a median number of prior therapies of 4 (range: 3-6) and significant lymphadenopathy in all patients except one (89%). Prior therapy included ibrutinib and venetoclax in 8/9 (89%) and 5/9 (56%) patients, respectively (**Table 1**).

**Table 1:** Patients’ baseline characteristics, toxicity and outcome of patients with CLL, with or without RT, who were included in the ARI-0001 program (CART19-BE-01 trial or compassionate use).

Pt	Sex	Age	Prior therapy	IGHV	Genomic aberrations	IPI	Dose infused (x10^6^/kg)	CRS grade	ICANS grade	Response (iwCLL)	Outcome
1	F	53	FCR, BR, I, V, IdR, O	U	13q-	–	1	1	0	PR	Alive and disease-free (45.4+ mo)
2	M	57	BR, R-CHOP, I, IdR, V	U	17p-, *NOTCH1* and *TP53* mutations	L	5	1	0	CR	Alive and disease-free (26.7+ mo)
3	F	51	R-CHOP, R-ESHAP, RIE	NA	17p-, t(11;21)	HI	–	–	–	–	Died while awaiting infusion (delayed due to COVID-19 pandemic)
4	M	72	Chl, BR, I, CHOP	U	*TP53* mutation	HI	0.5	2	0	CR	Alive and disease-free (12.5+ months)
5	M	74	I, IdR, V	U	13q-, 17p-, CK	H	5	0	0	SD	Died of CD19- relapse (7.0 mo)
6*	M	64	R-HyperCVAD, R-CHOP, I,	M	t(8;22), *MYC* rearrangement	H	0.5	1	0	PR	Died of CD19- relapse (3.7 mo)
7	F	65	FCR (x2), I	NA	13q-, 17p- and *TP53* mutation	–	0.4	3	0	CR	Alive and disease-free (4.2+ mo)
8	F	47	FCR, I, V, alloHCT	NA	13q-, 11q-, CK and *TP53* mutation	HI	1	1	0	CR	Alive and disease-free (1.4+ mo)
9	M	58	FCR, I, V	NA	13q-, 11q-, 17p-, CK	–	1	1	0	PR	Alive and disease-free (1.2+ mo)

Patients with concomitant RT are highlighted in grey.

Pt, patient; F, female; M, male; FCR, fludarabine, cyclophosphamide and rituximab; BR, bendamustine and rituximab; I, ibrutinib; V, venetoclax; O, obinutuzumab; IdR, idelalisib and rituximab; R-CHOP, rituximab, cyclophosphamide, adriamycin, vincristine and prednisone; R-ESHAP, rituximab, etoposide, cytarabine, cisplatin and methyl-prednisolone; RIE, rituximab, etoposide and ifosfamide; Chl, chlorambucil; CHOP, cyclophosphamide, adriamycin, vincristine and prednisone; R-HyperCVAD, rituximab, cyclophosphamide, adriamycin, vincristine and dexamethasone; alloHCT, allogeneic hematopoietic cell transplantation; IGHV, immunoglobulin heavy chain gene variable region; U, unmutated; M, mutated; NA, not available; CK, complex karyotype; IPI, international prognostic index (referred to the diffuse large B-cell lymphoma); L, low risk; HI, high-intermediate risk; H, high risk; CRS, cytokine release syndrome; ICANS, immune effector-cell associated neurotoxicity syndrome; CR, complete response; PR, partial response.

*This patient was diagnosed with both chronic lymphocytic leukemia and B-cell prolymphocytic leukemia (B-PLL). Richter’s transformation (in the form of plasmablastic lymphoma) occurred in the B-PLL clone. Blood and marrow evaluation documented a MRD-negative CR for both CLL and B-PLL.

Besides patient 3, who was due cell infusion in April 2020 and never received it owing to the temporary suspension of our program caused by COVID-19, the remaining eight patients received ARI-0001 cells after lymphodepletion (7). The median vein-to-vein time was 27 (range: 22-81) days. The target dose was 1 x10^6^ and 5 x10^6^ ARI-0001 cells/kg for patients with CLL and CLL/RT, respectively (7), but two patients with CLL/RT only received 10-40% (0.4-0.5 x10^6^ cells/kg), as per protocol, due to cytokine release syndrome (CRS) requiring treatment with tocilizumab (7).

There was no procedure-related mortality in this cohort. The CRS rate was 87.5% (95% confidence interval [CI]: 47-99%), with a grade ≥3 CRS rate of 12.5% (95% CI: 0.3-53%). Three patients required tocilizumab, and none required corticosteroids. The only patient who developed grade 3 CRS was one of the patients who did not receive the full dose, as per protocol. ICANS was not observed, whilst grade 4 neutropenia was documented in 7/8 (87.5%) patients for a median of 11 (range: 6-40) days. Grade 4 thrombocytopenia was observed in 3/8 (37.5%) patients. Four patients suffered from grade ≥3 infections: two episodes of gastroenteritis by *Campylobacter coli* and *Campylobacter jejuni* and two occurrences of urinary tract infection by *Escherichia coli* and *Klebsiella pneumoniae*.

**Figure 1:**
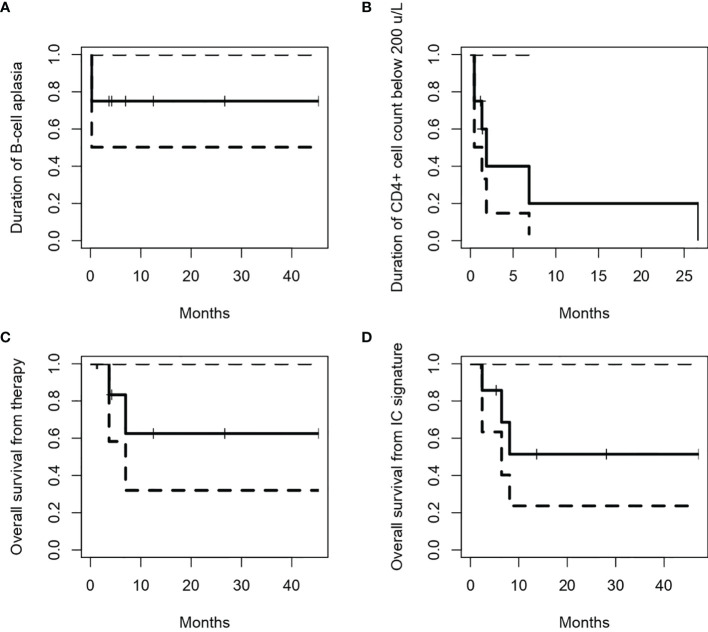
Duration of B-cell aplasia **(A)** and CD4^+^ cell counts below 200/µL **(B)** in patients who received ARI-0001 cell therapy. Overall survival from cell infusion **(C)** or signature of the informed consent **(D)**. Panels **(A–C)** only include patients who received therapy, while panel **(D)** include all patients included in the program (intention-to-treat).

**Figure 2:**
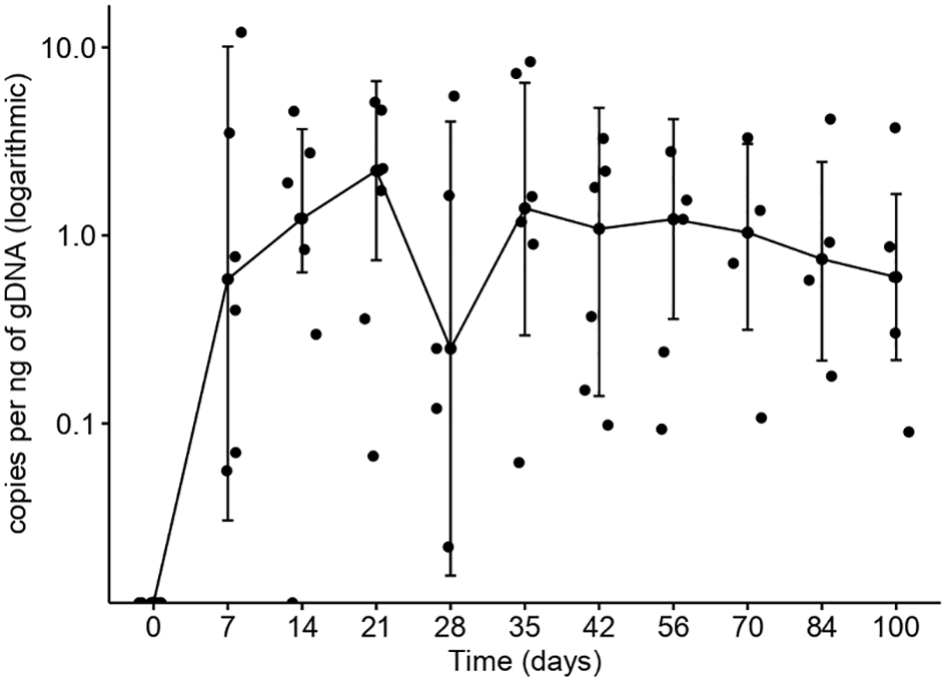
ARI-0001 expansion over time, calculated by quantitative PCR, in patients with CLL/RT. Lines represent median values.

All infused patients experienced absolute B-cell aplasia (BCA), whose median duration had not been reached (75% [95% CI: 50-100%] at 1 year, **Figure 1A**). Four (50%) patients had IgG determinations below 3 g/L (two of them already at screening), and five (56%) have required frequent Ig replacement. All patients had CD4+ T-cell counts below 200/µL, which recovered (>200/µL) at a median of 1.98 months after the ARI-0001 cell infusion (**Figure 1B**). ARI-0001 cell expansion, as measured by quantitative PCR, is displayed in **Figure 2**.

**Figure 3:**
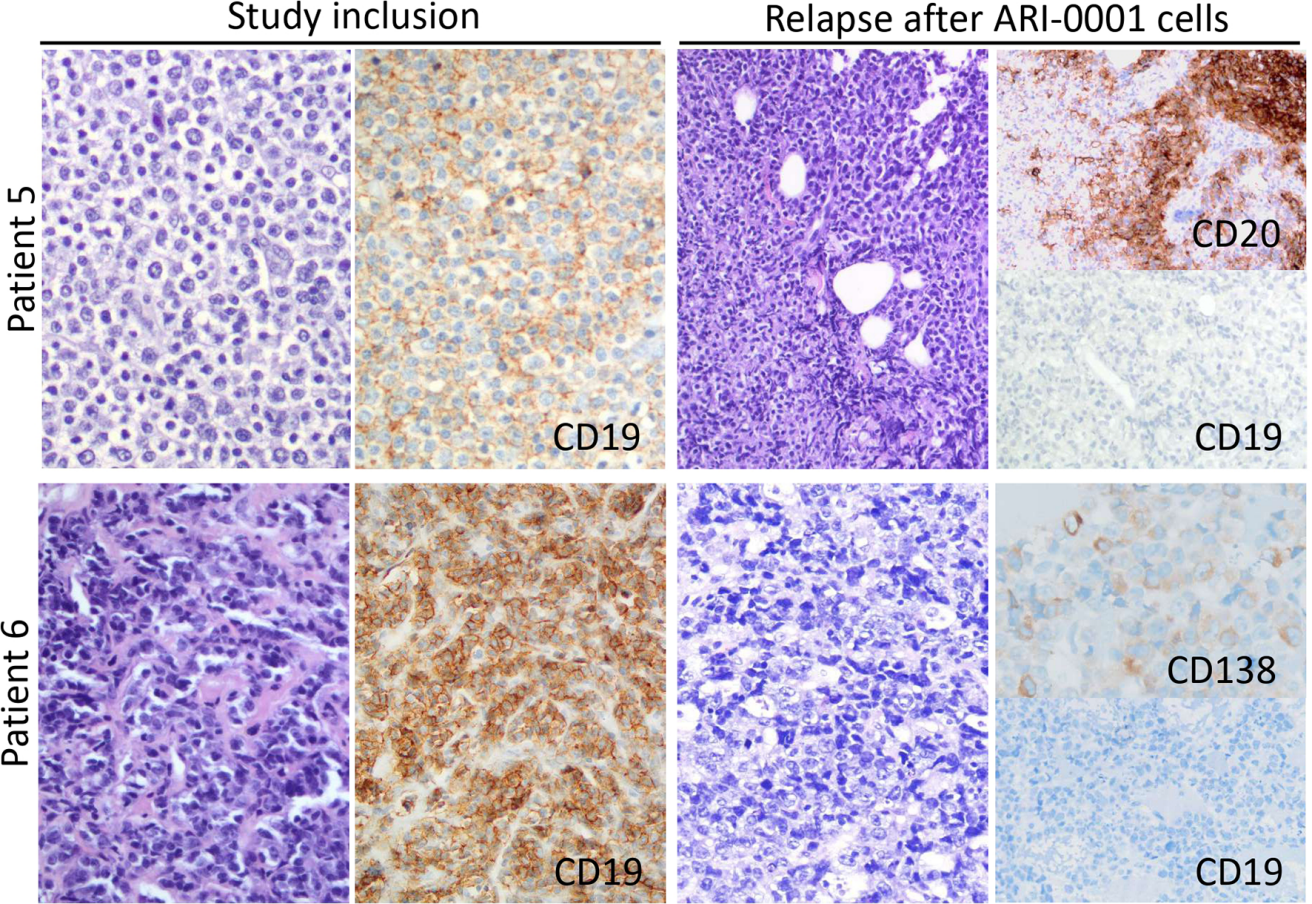
Lymph node morphology and phenotype observed in patients 5 and 6 at both study inclusion and relapse after treatment with ibrutinib-primed ARI-0001 cells. In both cases, CD19 expression was lost upon relapse.

Seven (87.5%; 95% CI: 47-99%) patients responded according to iwCLL/Lugano criteria (CR, n = 4; PR, n = 3) (10), while one patient with RT remained with stable disease. Measurable-residual disease (MRD) was undetectable in the peripheral blood and bone marrow of all patients. With a median follow-up of 5.6 (range, 1.2-45.3) months, two patients with RT experienced a CD19-negative relapse in the lymph nodes 2.1 and 3.0 months after cell infusion (**Figure 3**). The 2-year OS was 62.5% (95% CI: 32-100%) from ARI-0001 cell infusion and 51.4% (95% CI: 24-100%) from inclusion in the program (this last figure includes all nine patients) (**Figures 1C, D**).

## Discussion

Our results were comparable to other clinical trials and small series of patients with CLL/RT treated with similar CART19 products (12, 13), which have revealed ORRs around 38-82%, with CRRs around 20-45% (14–17). Of note, patients with RT were excluded from two pivotal trials performed in patients with DLBCL (18, 19) and, in a third trial, five patients with RT were included but their specific clinical outcome was not reported (20).

Since ibrutinib improves preclinical CART19 efficacy (21, 22), a number of clinical trials are evaluating the concomitant use of ibrutinib and CART19 cells in patients with CLL (13, 23, 24). In our experience, ARI-0001 cells could be manufactured for all patients and infused in 89% of them. A very unfortunate patient died before cell infusion, not because of excessive manufacturing time but because of the COVID-19 pandemic. Robust *in vivo* ARI-0001 cell expansion and persistent absolute B-cell aplasia was observed in most patients (**Figures 1**, **2**), this leading to undetectable MRD in the peripheral blood and bone marrow of all patients treated, even in those achieving a PR or stable disease in the lymph nodes. Unfortunately, two patients with RT have relapsed with CD19-negative disease despite no prior anti-CD19 therapy. This suggests that, perhaps, ARI-0001 cell therapy works better before RT occurs. However, we would still recommend ARI-0001 cell therapy for patients with RT since 3/5 patients remain in CR 1.4, 12.5 and 26.7 months after therapy.

In terms of toxicity, the fractionated administration of ARI-0001 cells appeared safe, with no cases of procedure-related mortality. The grade ≥3 CRS rate was 12.5% and there were no occurrences of ICANS. Despite the prolonged absolute B-cell aplasia and frequent grade 4 neutropenia, the incidence of severe infections was not greater compared to similar trials (13, 15–17), and the brief exposition to ibrutinib had a minor impact on toxicity.

In conclusion, our results suggest that the administration of ARI-0001 cells is feasible, safe, and effective in patients with high-risk CLL or RT. The role of concomitant ibrutinib therapy is still debated, but we hope that ongoing and future clinical trials will help us answer this question.

## Data Availability Statement

The raw data supporting the conclusions of this article will be made available by the authors, without undue reservation.

## Ethics Statement

The studies involving human participants were reviewed and approved by the Hospital Clínic de Barcelona. The patients/participants provided their written informed consent to participate in this study.

## Author Contributions

JD, MJ, ÁU-I, and JE designed the clinical trial. ME-R, MP, DB-R, and MJ were responsible for ARI-0001 cell production and monitoring after infusion. VO-M, NM-C, LM, EG, JC, AM-R, AL-G, and JD looked after the patients during the study. GF, OB and EC were responsible for pathological evaluation of lymph node biopsies. JC and ML were responsible for leukocytoapheresis. NV was responsible for immunophenotypic evaluation of peripheral blood and bone marrow samples. JD was responsible for the statistical analysis. VO-M and JD wrote the manuscript, which was approved by all authors. All authors contributed to the article and approved the submitted version.

## Conflict of Interest

VO-M: Consultant or advisory role (Kite Gilead, Celgene, Novartis), travel grants (Kite Gilead, Celgene, Novartis, Roche, Takeda, Janssen), honoraria (Kite Gilead). EG: Consultant or advisory role (Kite Gilead, Janssen, Genmab), research funding (Kite Gilead, Janssen, Roche). AM-R: Consultant or advisory role (Bristol Myers Squibb, Abbvie), travel grants (Kite Gilead, Roche, Takeda, Janssen, Abbvie), honoraria (Abbvie). ML: Honoraria (Grifols, Fresenius Kabi), research funding (Terumo BCT, Maco-Pharma). JE: Consultant or advisory role (Abbvie, Novartis, Celgene, Astellas, Jazz, Daiichi Dankyo, Roche, Amgen, Pfizer), travel grants (Celgene, Roche, Astellas, Daiichi Dankyo), research funding (Novartis, Celgene). AL-G: Consultant or advisory role (Roche, Kite Gilead, Celgene/Bristol-Myers, Incyte), honoraria (Roche, Novartis, Takeda, Bayer, Sandoz, Kern), research grants (Roche, Kite Gilead, Celgene/Bristol-Myers, Novartis, Incyte, Janssen, Pfizer, Takeda). ÁU-I: Consultant or advisory role (Kite Gilead, Celgene, Miltenyi), travel grants (Kite Gilead, Celgene). MJ: Consultant or advisory role (Kite Gilead, Grifols), honoraria (Kite Gilead, Grifols).

The remaining authors declare that the research was conducted in the absence of any commercial or financial relationships that could be construed as a potential conflict of interest.

## Publisher’s Note

All claims expressed in this article are solely those of the authors and do not necessarily represent those of their affiliated organizations, or those of the publisher, the editors and the reviewers. Any product that may be evaluated in this article, or claim that may be made by its manufacturer, is not guaranteed or endorsed by the publisher.
